# Nurses' and auxiliary nurse midwives' adherence to essential birth practices with peer coaching in Uttar Pradesh, India: a secondary analysis of the BetterBirth trial

**DOI:** 10.1186/s13012-019-0962-7

**Published:** 2020-01-03

**Authors:** Rose L. Molina, Brandon J. Neal, Lauren Bobanski, Vinay Pratap Singh, Bridget A. Neville, Megan Marx Delaney, Stuart Lipsitz, Ami Karlage, Mrunal Shetye, Katherine E. A. Semrau

**Affiliations:** 1 Division of Global and Community Health, Department of Obstetrics and Gynecology, Beth Israel Deaconess Medical Center, 330 Brookline Ave, Boston, MA 02215 USA; 2 Harvard Medical School, 25 Shattuck St, Boston, MA 02115 USA; 3 Ariadne Labs, 401 Park Drive, 3rd Floor East, Boston, MA 02215 USA; 4 Community Empowerment Lab, 26/11 Wazir Hasan Road, Lucknow, Uttar Pradesh 226001 India; 5 Bill & Melinda Gates Foundation, Capital Court, 5th Floor, Olof Palme Marg, Munirka, Delhi, India; 6 Division of Global Health Equity, Department of Medicine, Brigham and Women’s Hospital, 75 Francis St, Boston, MA 02115 USA

**Keywords:** Birth attendant, Competency, Coaching, Childbirth, Uttar Pradesh

## Abstract

**Background:**

The BetterBirth trial tested the effect of a peer coaching program around the WHO Safe Childbirth Checklist for birth attendants in primary-level facilities in Uttar Pradesh, India on a composite measure of perinatal and maternal mortality and maternal morbidity. This study aimed to examine the adherence to essential birth practices between two different cadres of birth attendants—nurses and auxiliary nurse midwives (ANMs)—during and after a peer coaching intervention for the WHO Safe Childbirth Checklist.

**Methods:**

This is a secondary analysis of birth attendant characteristics, coaching visits, and behavior uptake during the BetterBirth trial through birth attendant surveys, coach observations, and independent observations. Descriptive statistics were calculated overall, and by staffing cadre (staff nurses and ANMs) for demographic characteristics. Logistic regression using the Pearson overdispersion correction (to account for clustering by site) was used to assess differences between staff nurses and ANMs in the intervention group during regular coaching (2-month time point) and 4 months after the coaching program ended (12-month time point).

**Results:**

Of the 570 birth attendants who responded to the survey in intervention and control arms, 474 were staff nurses (83.2%) and 96 were ANMs (16.8%). In the intervention arm, more staff nurses (240/260, 92.3%) received coaching at all pause points compared to ANMs (40/53, 75.5%). At baseline, adherence to practices was similar between ANMs and staff nurses (~ 30%). Overall percent adherence to essential birth practices among ANMs and nurses was highest at 2 months after intervention initiation, when frequent coaching visits occurred (68.1% and 64.1%, respectively, *p* = 0.76). Practice adherence tapered to 49.2% among ANMs and 56.1% among staff nurses at 12 months, which was 4 months after coaching had ended (*p* = 0.68).

**Conclusions:**

Overall, ANMs and nurses responded similarly to the coaching intervention with the greatest increase in percent adherence to essential birth practices after 2 months of coaching and subsequent decrease in adherence 4 months after coaching ended. While coaching is an effective strategy to support some aspects of birth attendant competency, the structure, content, and frequency of coaching may need to be customized according to the birth attendant training and competency.

**Trial registration:**

ClinicalTrials.gov: NCT2148952; Universal Trial Number: U1111–1131-5647.

Contributions to the literature

- This study provides specific details about a coaching program around the WHO Safe Childbirth Checklist and subsequent adherence to essential birth practices according to birth attendant cadre (staff nurses vs. auxiliary nurse midwives).
- This study describes implementation science methods for evaluating a birth attendant coaching program at scale in Uttar Pradesh, India.
- This study reports the occurrence of unqualified health facility personnel who deliver childbirth care at primary-level facilities in Uttar Pradesh, India.
- This study describes the demographic characteristics of birth attendants in primary-level facilities in Uttar Pradesh, India.

## Background

In response to Millennium Development Goal 5 to reduce the maternal mortality ratio by 75% [1], there has been a global push to increase the proportion of women who deliver in facilities with skilled birth attendants. In 2014, 71% of all births occurred with skilled birth attendants compared to 59% of all births in 1990 [2]. Despite this increase, maternal mortality has not declined as quickly as anticipated [3, 4]. Therefore, global focus has begun shifting to the quality of care delivered in facilities [3, 4]. Factors that influence the quality of care in facility-based childbirth for low- and middle-income countries (LMICs) include training and supervision, staff numbers and workloads, salaries and living conditions, and functionality of the health system [3]. Of these factors, inadequate clinical training and supervision of skilled birth attendants have emerged as fundamental concerns [3].

To address concerns about skilled birth attendants' competencies, multiple global agencies including the World Health Organization (WHO), United Nations Population Fund (UNFPA), United Nations Children’s Fund (UNICEF), International Confederation of Midwives (ICM), and International Federation of Gynecology and Obstetrics (FIGO) recently published a new definition for skilled birth attendants [5]. The revised definition emphasizes competency in 1) providing evidence-based and dignified care to women and newborns, 2) facilitating physiologic labor and birth and promoting a positive birth experience, and 3) identifying and managing or referring women and newborns with complications [5]. This definition of competency specifies required knowledge, skills, and behaviors rather than educational, training, or experience-based qualifications. We lack data about ongoing birth attendant competency after completion of different professional training programs in many LMICs where there are often no continuing professional training requirements. In Uttar Pradesh, India, auxiliary nurse midwives (ANMs) receive 2 years of training while nurses receive 4 years of training with varying duration of clinical supervision [6]. Due to the differences in training and work experience between ANMs and nurses, performance improvement strategies may require customization for each cadre of birth attendants.

Coaching is one method to improve clinical performance, including individualized feedback during real-time observation of specific behaviors to enhance performance [7]. In the BetterBirth trial, we designed a coaching intervention to overcome the “know–do” gap by identifying and resolving opportunity, ability, motivation, and supply (OAMS) barriers in birth attendant adherence to essential birth practices codified in the WHO Safe Childbirth Checklist [8–10]. The BetterBirth trial demonstrated an increase in adherence to essential birth practices among birth attendants but no corresponding improvement in maternal or neonatal outcomes in frontline childbirth facilities [11]. Traditional facility-level indicators were not associated with maternal or neonatal outcomes [12], but differences in birth attendant training have not been evaluated with regard to response to coaching and adherence to essential birth practices. This study examines the characteristics of the two cadres of birth attendants (nurses and ANMs), the amount of coaching each cadre received, and their adherence to essential birth practices over 12 months as an assessment of their response to coaching.

## Methods

This study is a secondary analysis of birth attendant characteristics, coaching visits, and behavior uptake during the BetterBirth trial. The BetterBirth trial was a matched-pair, cluster-randomized controlled trial studying the effect of a coaching-based implementation of the WHO's Safe Childbirth Checklist on a composite measure of perinatal mortality, maternal mortality, and severe maternal morbidity, all within 7 days after delivery (ClinicalTrials.gov: NCT2148952; Universal Trial Number: U1111–1131-5647). The trial took place in 120 public primary and community health centers throughout the state of Uttar Pradesh in northern India, described in detail elsewhere [11, 13].

The WHO Safe Childbirth Checklist included 28 evidence-based, essential birth practices that should be performed for every woman and newborn during labor and delivery [14]. The Checklist arranged the 28 practices into 4 pause points (or moments of care): on admission, just before pushing or cesarean, within 1 h after delivery, and on discharge.

The coaching-based implementation of the WHO Safe Childbirth Checklist took place between December 2014 and September 2016 and has been described in detail elsewhere [8, 9]. Coaches were nurses with training in childbirth care and were recruited from the same geographic hub as their facility assignments [8]. They received extensive training and support to carry out their responsibilities, which included motivating birth attendants, observing and providing feedback to birth attendants, and problem solving with birth attendants [8]. For example, coaches worked with nurses and ANMs to identify and solve barriers in adherence to the practices on the checklist. The frequency of the coaching visits tapered over the course of 8 months: in months 1–4, coaching visits occurred twice weekly; in months 5–6, coaching visits occurred weekly; in month 7, coaching visits occurred every other week; and in month 8, coaching visits occurred once. Each coaching visit was expected to last 7–8 h. Any coaching visit that lasted less than 4 h was excluded from analysis.

We utilized three different methods of data collection in this secondary analysis: birth attendant survey, coach observation, and independent data collector observation.

### Birth attendant survey

We conducted this survey by phone after completion of the main trial. We asked all birth attendants who had participated in the study in both the intervention and control arms to share their demographic information including education, training, experience, and age. We calculated descriptive statistics overall and by staffing cadre (staff nurses and ANMs) for these characteristics and presented them as frequencies and proportions or as a median with interquartile range, as appropriate.

### Coach observations

**Fig. 1:**
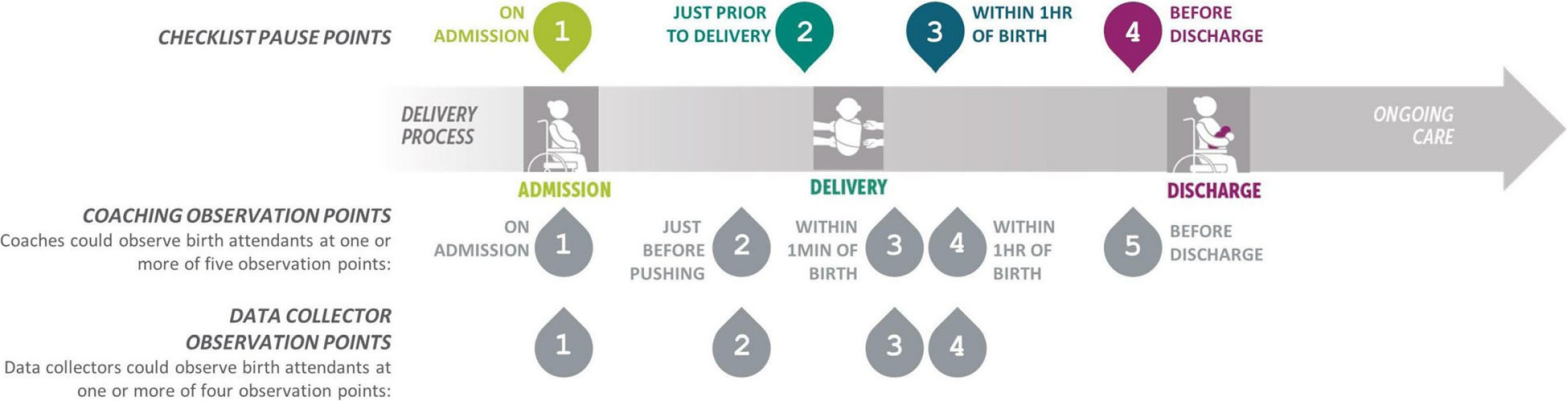
Birth attendant observation points

Coaches observed birth attendant adherence to essential birth practices only in the intervention arm. Birth attendants could be observed for 1 or more of 5 different observation points: 1) on admission, 2) just before pushing, 3) within 1 min of delivery, 4) within 1 h of delivery, and 5) before discharge (Fig. 1). For each birth attendant in the intervention arm, we recorded the occurrence of coaching and the number of days coached at each observation point and overall. We reported these data by staffing cadre (staff nurses and ANMs) as counts with percentages and as medians with interquartile ranges, respectively.

### Independent data collector observations

Independent observers were nurses trained in childbirth care who received extensive training to record behavioral data with a standardized tool [9]. Throughout the trial—at 0, 2, 6, and 12 months after the start of the coaching intervention—independent observers went to a subset of the intervention and control sites to document adherence to the essential birth practices on the Checklist. At 0 and 6 months after coaching started, data collectors observed 5 sites from each arm. At 2 and 12 months after coaching started, data collectors observed 15 sites from each arm, including the 5 sites from each arm observed at 0 and 6 months. Data collectors could observe a birth attendant at one or more of the following observation points: (1) on admission, (2) just before pushing, (3) within 1 min of delivery, and (4) within 1 h after delivery. Independent observers did not document practices on discharge, as women often left the facility very soon after delivery (Fig. 1). Additionally, for each observation point, data collectors recorded the number and type of birth attendants present. The independent observer was able to select from the following, non-mutually exclusive, choices: ANM, staff nurse, doctor, and other. Independent observers had the option of entering free text comments, which at times included specific reference to non-clinical personnel who participated in delivery care (such as cleaners or *dais*). For analysis, we created four mutually exclusive categories to define who delivered the woman: doctor only, staff nurse and ANM, staff nurse only, or ANM only. Due to the small number of observations, we dropped deliveries attended by the doctor only or by the staff nurse and ANM jointly from further analyses. We stratified adherence to essential birth practices by observational time point and presented it by arm and staffing cadre. We also graphed adherence to four essential birth practices and the frequency of Checklist use by cadre, trial arm, and time point.

Using observations from independent data collectors, we created a flow chart of complication management for elevated blood pressure. At 12 months after coaching began, when independent data collectors observed birth attendants taking maternal blood pressure, they also recorded the corresponding values. We identified high blood pressure as diastolic blood pressure greater than or equal to 110 mmHg. Independent observers also recorded whether magnesium sulfate was appropriately administered to individuals with high blood pressure.

### Statistical analysis

The analyses of the birth attendant survey, coach observations, and data collector observations were descriptive with results presented in proportions and medians as described above. The main analysis describing relationships between birth attendant cadres and behaviors used data from the independent observer database. In particular, we calculated an overall percent of behavior adherence for ANM only and staff nurse only in both arms stratified by time point from initiation of coaching. We used logistic regression using the Pearson overdispersion correction (to account for clustering by site) to assess differences between staff nurses and ANMs in the intervention group at 2 and 12 months after coaching began. We did not conduct statistical testing for 0 and 6 months after coaching began due to the limited number of observations from only 5 sites.

The BetterBirth trial protocol was approved by ethics review boards at Community Empowerment Lab, Jawaharlal Nehru Medical College, Harvard T.H. Chan School of Public Health, Population Services International, the WHO, and the Indian Council of Medical Research. The protocol was reviewed and reapproved on an annual basis. For coaching, each facility and birth attendant formally agreed to participate in the BetterBirth program as a quality improvement initiative at the beginning of the intervention. For independent observation, each facility and birth attendant consented to participate in the study. Prior to each observation, independent observers verbally confirmed that the birth attendant agreed to be observed. Women who presented for labor and delivery signed written consent to have independent observers present during their care.

## Results

In total, 647 birth attendants from both intervention and control sites were contacted for the survey. Of the 647 birth attendants, 610 (94.3%) responded, and 37 (5.7%) did not respond. Among the 610 respondents, 40 were Lady Medical Officers (the official term for female physicians in India), who we excluded in the analysis of nurses and ANMs. Therefore, 570 birth attendants were included in our analysis, representing 88.1% of the total birth attendants surveyed. The majority of respondents were staff nurses (*n* = 474, 83.2%), and the remaining respondents were ANMs (*n* = 96, 16.8%; Table 1). This sample was consistent with the distribution of births attended by each birth attendant cadre. Among the 161,157 births recorded during the trial, 130,845 (81.2%) were attended by staff nurses and 29,861 (18.5%) were attended by ANMs. Compared to ANMs, staff nurses were younger (median age 32 vs. 52.5 years), had more education (48.3 vs. 23.3% held Bachelors or Master's degree), and had less work experience (median 5 vs. 28.3 years). Less than half (47%) of both staff nurses and ANMs in the BetterBirth facilities in Uttar Pradesh completed the skilled birth attendant training required by the state government.

**Table 1:** Birth attendant demographics by staffing cadre in the 120 health facilities in the BetterBirth trial (intervention and control)

	ANM *n* = 96	Staff nurse *n* = 474	Total *N* = 570
Intervention	43 (44.8)	240 (50.6)	283 (49.6)
Control	53 (55.2)	234 (49.4)	287 (50.4)
SBA training	44 (45.8)	223 / 472 (47.3)	267 / 568 (47.0)
Highest nursing course			
2 years	91 (94.8)	4 (0.8)	95 (16.7)
3 years	5 (5.2)	464 (97.9)	469 (82.3)
4 years	0 (0.0)	6 (1.3)	6 (1.1)
Highest education			
Bachelor/Master degree	20 / 86 (23.3)	188 / 389 (48.3)	208 / 475 (43.8)
Secondary school	66 / 86 (76.7)	201 / 389 (51.7)	267 / 475 (56.2)
Age (years)^a^	96, 52.5 (43.5–57)	472, 32 (28–38)	568, 33 (28.5–44)
Years since last training^a^	43, 3 (2–7)	219, 3 (2–7)	262, 3 (2–7)
Years of birth attendant experience^a^	96, 28.3 (10–32)	472, 5 (3–9)	568, 6 (4–10.6)

Denominators reported where different from total

^a^*n*, median (Q1–Q3)

Among the 6562 births for which an independent data collector documented at least one observation point, 328 births (5.0%) were attended by non-clinical facility staff (such as cleaners). Of the 328 births attended by non-clinical facility staff, 129 (39.3%) occurred in the control arm and 199 (60.7%) occurred in the intervention arm.

In intervention facilities, all staff nurses and ANMs who responded to the survey received coaching at least once (Table 2). Staff nurses received a median of 13 coaching visits (IQR 8, 17), and ANMs received a median of 6 coaching visits (IQR 4, 11). More staff nurses (92.3%) received coaching at all 5 pause points compared to ANMs (75.5%).

**Table 2:** Birth attendant coaching dose received stratified by cadre in the 60 intervention sites of the BetterBirth trial

	ANM *n* = 53 (%); median (Q1, Q2)	Staff nurse *n* = 260 (%); median (Q1, Q2)
Received coaching point 1		
Proportion	45 (84.9)	251 (96.5)
Number of days^*^	3.0 (1, 5)	7.0 (3, 9)
Received coaching point 2		
Proportion	47 (88.7)	252 (96.9)
Number of days^*^	3.0 (2, 6)	7.0 (4, 11)
Received coaching point 3		
Proportion	49 (92.5)	251 (96.5)
Number of days^*^	5.0 (2, 8)	8.0 (4.5, 12)
Received coaching point 4		
Proportion	48 (90.6)	252 (96.9)
Number of days^*^	4.0 (2, 7)	7.0 (4, 11)
Received coaching point 5		
Proportion	49 (92.5)	254 (97.7)
Number of days^*^	5.0 (2, 8)	9.0 (6, 13)
Coached at least once	53 (100)	260 (100)
Received all 5 coaching points		
Proportion	40 (75.5)	240 (92.3)
Number of days^*^	6.0 (4, 11)	13.0 (8, 17)

*Median (Q1, Q2)

**Fig. 2:**
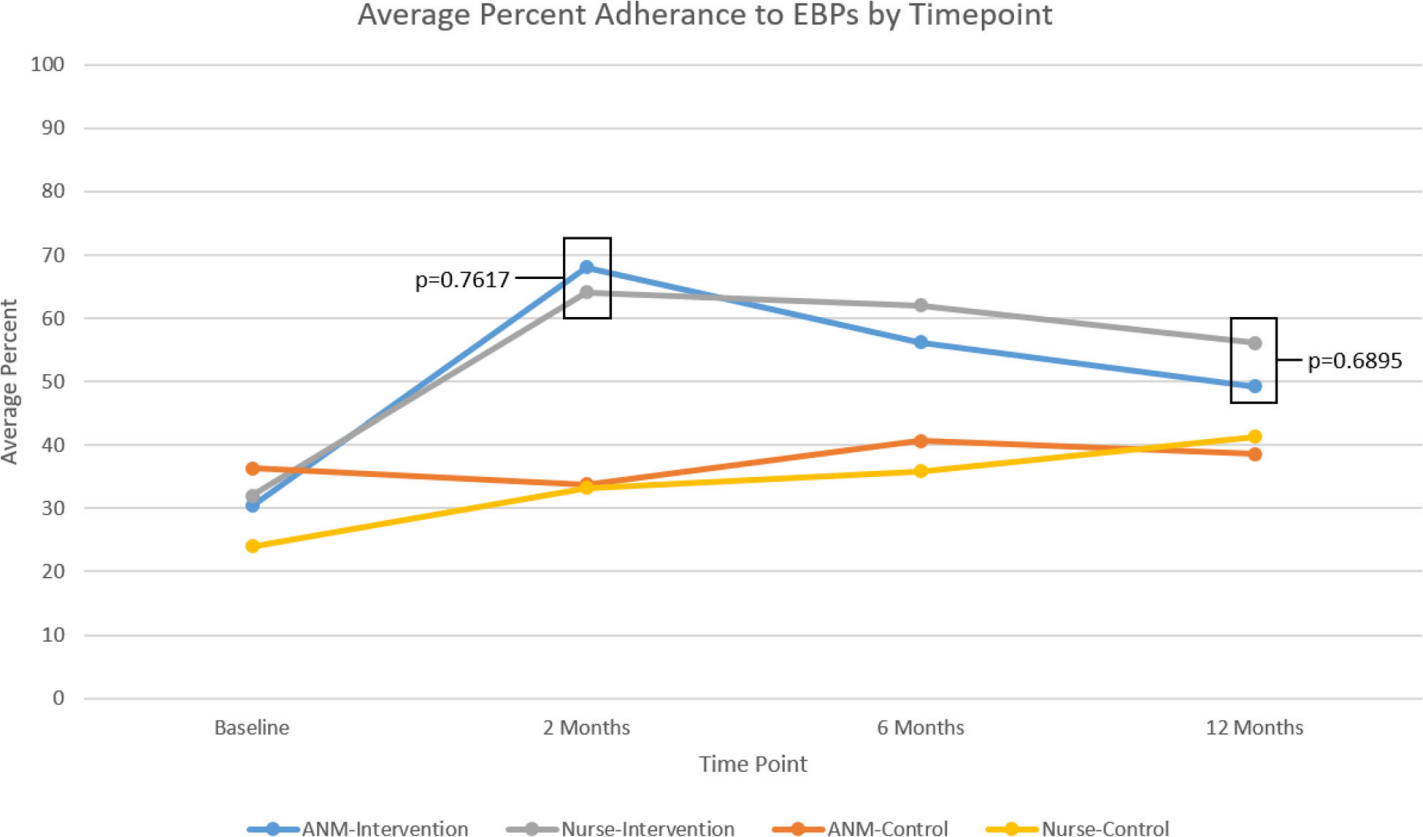
Average adherence to essential birth practices by time point and staffing cadre in 30 facilities in the BetterBirth trial

**Fig. 3:**
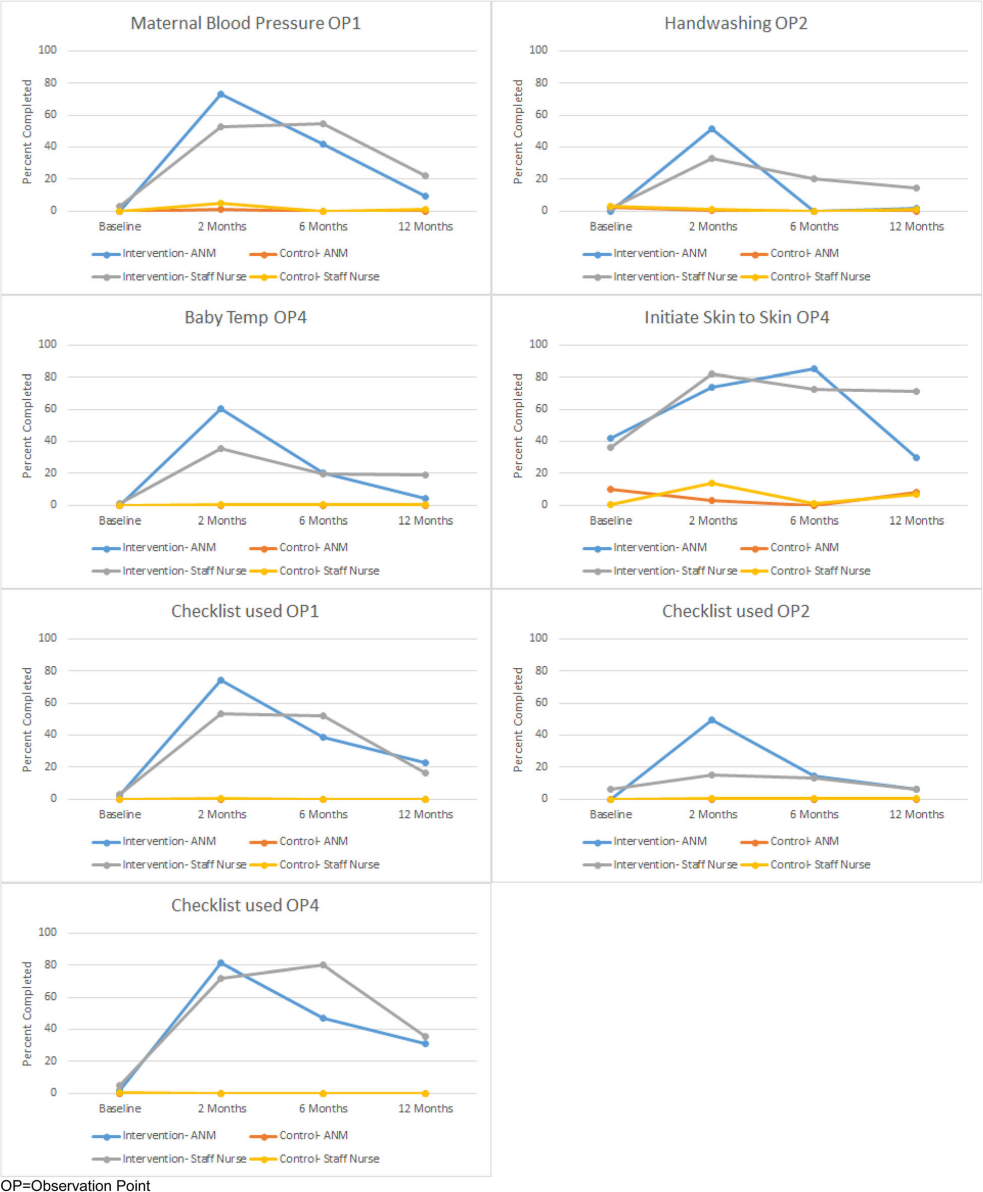
Select behaviors and checklist use during observation point (OP) by birth attendant cadre in the BetterBirth trial

Overall, staff nurses and ANMs in the intervention arm, who received coaching on the Checklist, adhered to more essential birth practices than those in the control arm, who did not receive coaching (Fig. 2, Additional file 5: Table S5). Baseline adherence to essential birth practices among staff nurses and ANMs was similar at approximately 30% (Fig. 2, Additional file 5: Table S5). Adherence to essential birth practices among ANMs and nurses was highest at 2 months after coaching began, when coaching visits were most frequent (68.1 and 64.1%, respectively, *p* = 0.76). At 12 months after coaching began (4 months after coaching ended), ANMs demonstrated 49.2% adherence to all essential birth practices, and nurses demonstrated 56.1% adherence (*p* = 0.69). However, they did not sustain the increased adherence to checking maternal blood pressure during admission, hand hygiene prior to delivery, and checking newborn temperature at 1 h after birth (Fig. 3). We selected these essential birth practices because they reflect application of knowledge and skills rather than verifying supplies. Staff nurses continued to initiate skin-to-skin contact after birth in intervention facilities at 12 months after coaching began (71.1 vs 29.8% among ANMs). Data on the adherence of each cadre to individual essential birth practices at all observation points are located in the Additional file 1: Table S1, Additional file 2: Table S2, Additional file 3: Table S3, and Additional file 4: Table S4.

After 2 months of coaching, independent observers noted that ANMs used the Checklist more frequently than staff nurses on admission (74.7 vs. 53.6%), just before pushing (49.7 vs. 15.1%), and within 1 h after birth (81.3 vs. 72.0%; Additional file 2: Table S2). After 12 months, both ANMs and staff nurses demonstrated much lower adherence to using the Checklist on admission (22.9 vs. 16.4%), just before pushing (6.0 vs. 5.9%), and within 1 h after birth (30.7 vs. 35.4%; Additional file 4: Table S4).

**Fig. 4:**
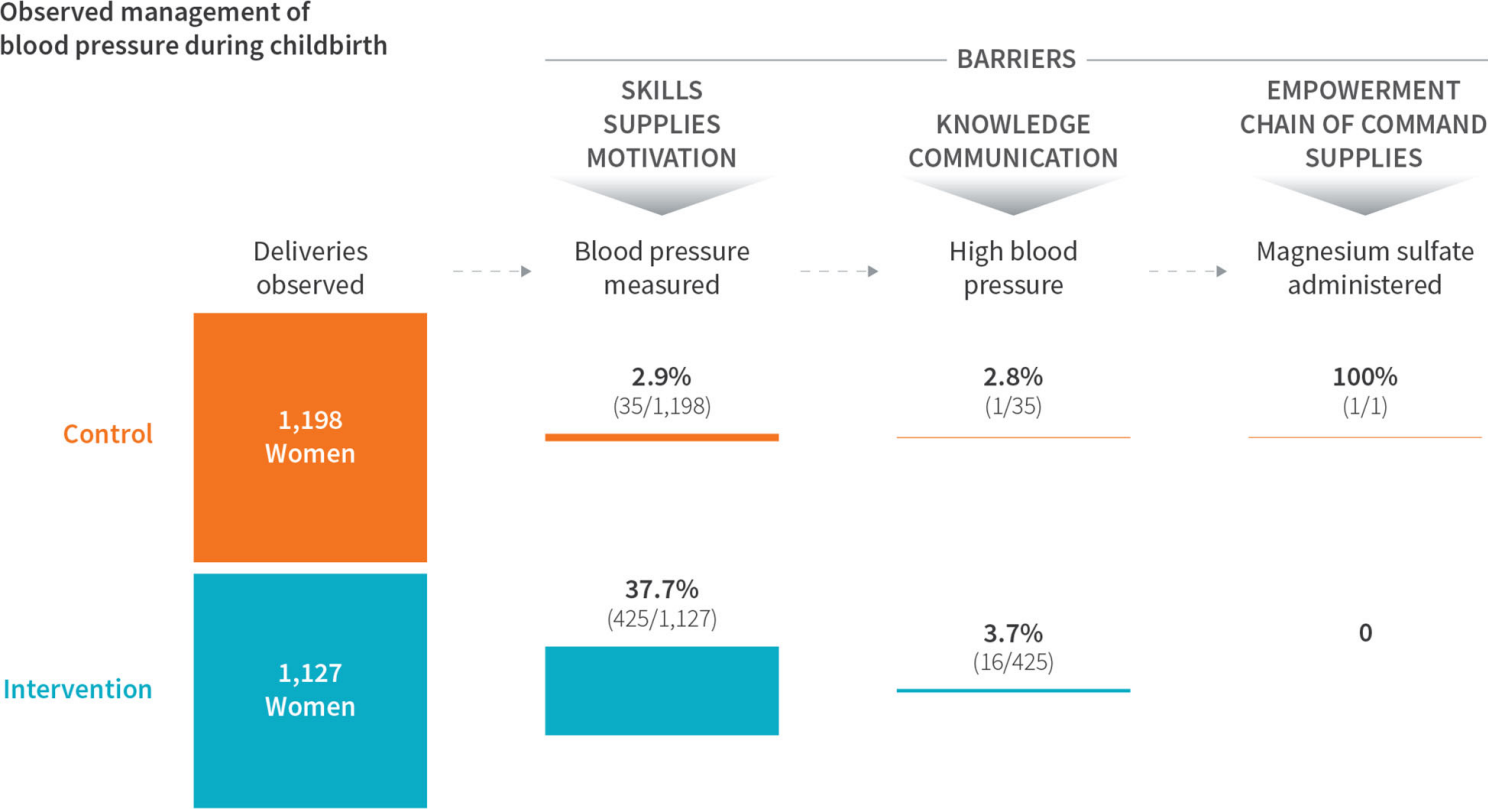
Complication management of elevated blood pressure in 30 facilities in the BetterBirth trial at the 12-month observation point

At 12 months after coaching began, independent observers recorded inadequate blood pressure management. While 37.7% of women had their blood pressure checked in intervention sites compared to 2.9% of women in control sites, only 1 of the 17 women with an abnormal diastolic blood pressure (>  110 mmHg) received magnesium sulfate as indicated (Fig. 4).

## Discussion

Birth attendant competency has gained global attention as a critical foundation for high-quality childbirth care [15]. The birth attendants in the BetterBirth trial generally fell into two categories: ANMs were older and had much more experience than nurses; however, nurses had more education, on average, than ANMs. Notably, less than half of both cadres received the formal Skilled Birth Attendant training, which is implemented through the National Health Mission to fill competency gaps among nurses and midwives who work in primary-level facilities [16].

Nurses were coached more frequently across all 5 observation points than ANMs. This unequal amount of coaching may reflect the staffing ratios in primary-level facilities where there are more nurses than ANMs available for coaching in general and specifically during daytime hours when coaching visits took place. Coaches may have felt more comfortable with nurses due to similar demographic characteristics such as age. It is important for coaching programs to reach all birth attendants, regardless of staffing availability, so that all can receive the benefit of individual observation and feedback to improve performance. Inter-professional differences between ANMs and nurses—such as age, education, and experience—should be considered when designing coaching programs because they may influence the frequency and content of coaching interactions.

At least 5% of deliveries were attended by non-clinical facility personnel, such as cleaners in the facility. While there is information regarding prevalence of unqualified personnel or traditional birth attendants who assist with deliveries in homes or communities [17], there is a lack of data regarding prevalence of unqualified personnel who participate in care in primary-level facilities. Several factors may explain the frequency of unqualified personnel attending births in facilities, including staff availability, birth attendant motivation, and women's preferences. For example, unqualified personnel may be the only ones available when all qualified birth attendants are busy with other patients when the patient census is high. Bias from both providers and patients may play a role in determining which patients receive attention from qualified versus unqualified staff.

In designing the BetterBirth coaching intervention, we assumed that all birth attendants met competency standards such as those set forth by the WHO based on abilities, skills, and knowledge rather than on educational or training qualifications alone. We did not measure birth attendant competency directly as a part of the BetterBirth trial. We designed the coaching intervention to help close the “know–do” gap by identifying and resolving opportunity, ability, motivation, and supply (OAMS) barriers—in short to facilitate skilled birth attendants practicing their competencies. The sharp increase in adherence to essential birth practices at 2 months after coaching began suggests that a coaching-based implementation of the Checklist can improve birth attendant behaviors, as measured by the ability to complete the steps of the Checklist, but such an improvement may require frequent, intensive coaching to sustain behavior change. Additionally, integration of new practices in health facilities often require extended efforts to sustain behavior change initially while more permanent integrating mechanisms take hold [18].

It is important to note that we measured adherence to essential birth practices, but not necessarily the quality of those practices performed. Complication management remained problematic, as demonstrated by the example of abnormal blood pressure management. In India, pre-service training for nurses and midwives may not provide sufficient clinical experience to meet international competency standards [16]. One cross-sectional survey among graduating students in 25 nursing and midwifery institutions demonstrated that 38–50% scored below the 50th percentile in all subscales for antepartum, intrapartum, postpartum, and newborn care [16]. Other studies have demonstrated variation in birth attendant competency [19, 20]. From the anecdotal experience of birth attendants and coaches in the BetterBirth trial, ongoing gaps in competency may be related to insufficient pre-service training and may be exacerbated by workplace factors such as chain of command regarding clinical decisions and supply gaps.

With early frequent coaching, adherence to essential birth practices increased, but was not sustained over time for many practices. The Government of India has partnered with Jhpiego in implementing Dakshata, a quality improvement program that focuses on boosting birth attendant competency in multiple states [21]. Program components include data collection about birth attendant competency and ongoing support through skills training. Other competency-based training programs concerning complication management such as postpartum hemorrhage have been developed and tested, but the long-term impact on skills retention and clinical outcomes remains unknown [22, 23]. Our study results suggest the importance of more frequent coaching visits especially in settings where competency may be limited. Additionally, coaching may require a tailored approach to meet individual birth attendants where they are regarding knowledge and skills and to account for differences among birth attendant cadres. Simulation-based training around complication management may be particularly useful in building skills around rare emergencies that preclude sufficient direct feedback through coaching [24–26].

### Limitations

These findings are subject to several limitations. As a secondary analysis of the BetterBirth trial, there may be measurement bias in identifying the specific cadre of birth attendant who was present during delivery. We assumed the birth attendant observed within 1 min of birth was also the birth attendant who attended the delivery. Additionally, it is possible that multiple birth attendants participated in the care of individual women over the course of their stay in the facility. These results relied upon independent observations, which may be vulnerable to the Hawthorne effect. However, we conducted observations repeatedly over several weeks, and long-term, repeated observations do not appear to affect behavior [27]. Further, we did find tapering of adherence to essential birth practices over time in intervention sites, which may suggest that the Hawthorne effect may not have lasted throughout the duration of the study. Additionally, we recognize the measurement bias in observers' notes regarding non-clinical facility personnel's participation in care delivery, which was likely an underestimate. Observers could not be blinded to which facilities received the intervention and which facilities did not, which may have influenced their reporting of who participated in care delivery.

## Conclusions

High-quality care during childbirth depends on competent birth attendants who are equipped with the human resources and supplies needed to manage complications. In the BetterBirth trial, we used coaching to boost adherence to key essential birth practices on the WHO Safe Childbirth Checklist. Overall, ANMs and staff nurses responded similarly to the coaching intervention with the greatest increase in adherence to essential birth practices after 2 months of coaching, with subsequent decrease in adherence after the coaching intervention was completed. Coaching is an effective strategy to support some aspects of birth attendant competency; however, the structure, content, duration, and frequency of coaching may need to be customized according to the birth attendant cadre and competency.

## Supplementary information

**Additional file 1: Table S1.** Baseline adherence to essential birth practices stratified by birth attendant cadre in 10 facilities in the BetterBirth trial.

**Additional file 2: Table S2.** 2-month adherence to essential birth practices stratified by birth attendant cadre in 30 facilities in the BetterBirth trial.

**Additional file 3: Table S3.** 6-month adherence to essential birth practices stratified by birth attendant cadre in 10 facilities in the BetterBirth trial.

**Additional file 4: Table S4.** 12-month adherence to essential birth practices stratified by birth attendant cadre in 30 facilities in the BetterBirth trial.

**Additional file 5: Table S5.** Average percent adherence to essential birth practices by time point and birth attendant cadre.

## Data Availability

The datasets generated and/or analyzed during the current study will be available in the Harvard Dataverse upon publication.

## Abbreviations

ANMs: Auxiliary nurse midwives
IQR: Inter-quartile range
LMICs: Low- and middle-income countries
OAMS: Opportunity, ability, motivation, supplies
WHO: World Health Organization
