# Supplementary material for: Nurses' and auxiliary nurse midwives' adherence to essential birth practices with peer coaching in Uttar Pradesh, India: a secondary analysis of the BetterBirth trial
Source: Implement Sci. 2020 Jan 3;15:1. doi: 10.1186/s13012-019-0962-7 (PMC6941293; doi:10.1186/s13012-019-0962-7)
Supplement: Supplementary file 1 — Additional file 1: Table S1. Baseline adherence to essential birth practices stratified by birth attendant cadre in 10 facilities in the BetterBirth trial. [file 13012_2019_962_MOESM1_ESM.docx]

**Additional file 1: Table S1. Baseline Adherence to Essential Birth Practices Stratified by Birth Attendant Cadre in 10 Facilities in the BetterBirth Trial**

|  | **Intervention** | | **Control** | |
| --- | --- | --- | --- | --- |
|  | **ANM (N=50)**  **n (%)** | **Staff Nurse (N=109)**  **n (%)** | **ANM (N=45)**  **n (%)** | **Staff Nurse (N=163)**  **n (%)** |
| Observations at OP1 | 50 | 109 | 45 | 163 |
| Mother's Temperature (OP1) | 1 (2) | 0 (0) | 0 (0) | 0 (0) |
| Mother's Blood Pressure (OP1) | 0 (0) | 3 (2.8) | 0 (0) | 0 (0) |
| Partograph Started (OP1) | 0 (0) | 0 (0) | 0 (0) | 0 (0) |
| Checklist Used (OP1) | 1 (2) | 3 (2.8) | 0 (0) | 0 (0) |
| Observations at OP2 | 50 | 103 | 41 | 136 |
| Oxytocin Administered (OP2) | 44 (88) | 73 (70.9) | 32 (78) | 105 (77.2) |
| Handwashing (OP2) | 0 (0) | 2 (1.9) | 1 (2.4) | 4 (2.9) |
| Prepare Clean Gloves (OP2) | 49 (98) | 74 (71.8) | 40 (97.6) | 99 (72.8) |
| Prepare Clean Towel (OP2) | 5 (10) | 22 (21.4) | 3 (7.3) | 10 (7.4) |
| Prepare Sterile Scissors / Blade (OP2) | 24 (48) | 30 (29.1) | 8 (19.5) | 34 (25) |
| Prepare Cord Ligature / Tie (OP2) | 41 (82) | 94 (91.3) | 41 (100) | 118 (86.8) |
| Prepare Mucus Extractor (OP2) | 46 (92) | 96 (93.2) | 41 (100) | 68 (50) |
| Prepare Bag & Mask (OP2) | 34 (68) | 59 (57.3) | 41 (100) | 124 (91.2) |
| Prepare Pads for Mother (OP2) | 20 (40) | 68 (66) | 33 (80.5) | 43 (31.6) |
| Checklist Used (OP2) | 0 (0) | 6 (5.8) | 0 (0) | 0 (0) |
| Observations at OP3 | 50 | 107 | 39 | 139 |
| Oxytocin Administered (OP3) | 5 (10) | 17 (15.9) | 24 (61.5) | 14 (10.1) |
| Other Uterotonic Administered (OP3) | 0 (0) | 0 (0) | 0 (0) | 0 (0) |
| Observations at OP4 | 60 | 108 | 41 | 160 |
| Baby Weight (OP4) | 24 (40) | 78 (72.2) | 38 (92.7) | 118 (73.8) |
| Baby Temperature (OP4) | 0 (0) | 1 (0.9) | 0 (0) | 0 (0) |
| Skin to Skin (OP4) | 25 (41.7) | 39 (36.1) | 4 (9.8) | 1 (0.6) |
| Skin to Skin 1 Hour (OP4) | 0 (0) | 6 (5.6) | 1 (2.4) | 0 (0) |
| Breastfeeding (OP4) | 24 (40) | 23 (1.3) | 2 (4.9) | 6 (3.8) |
| Checklist Used (OP4) | 1 (1.7) | 5 (4.6) | 0 (0) | 1 (0.6) |

OP=Observation Point
