# Supplementary material for: Nurses' and auxiliary nurse midwives' adherence to essential birth practices with peer coaching in Uttar Pradesh, India: a secondary analysis of the BetterBirth trial
Source: Implement Sci. 2020 Jan 3;15:1. doi: 10.1186/s13012-019-0962-7 (PMC6941293; doi:10.1186/s13012-019-0962-7)
Supplement: Supplementary file 2 — Additional file 2: Table S2. 2-month adherence to essential birth practices stratified by birth attendant cadre in 30 facilities in the BetterBirth trial. [file 13012_2019_962_MOESM2_ESM.docx]

**Additional file 2: Table S2. 2-month Adherence to Essential Birth Practices Stratified by Birth Attendant Cadre in 30 Facilities in the BetterBirth Trial**

|  | **Intervention** | | **Control** | |
| --- | --- | --- | --- | --- |
|  | **ANM** | **Staff Nurse** | **ANM** | **Staff Nurse** |
| Observations at OP1 | 166 | 758 | 165 | 761 |
| Mother's Temperature (OP1) | 124 (74.7) | 364 (48) | 0 (0) | 1 (0.1) |
| Mother's Blood Pressure (OP1) | 121 (72.9) | 399 (52.6) | 2 (1.2) | 36 (4.7) |
| Partograph Started (OP1) | 0 (0) | 8 (1.1) | 0 (0) | 0 (0) |
| Checklist Used (OP1) | 124 (74.7) | 406 (53.6) | 0 (0) | 1 (0.1) |
| Observations at OP2 | 175 | 704 | 169 | 728 |
| Oxytocin Administered (OP2) | 56 (32) | 215 (30.5) | 108 (63.9) | 535 (73.5) |
| Handwashing (OP2) | 90 (51.4) | 232 (33) | 1 (0.6) | 6 (0.8) |
| Prepare Clean Gloves (OP2) | 158 (90.3) | 639 (90.8) | 104 (61.5) | 498 (68.4) |
| Prepare Clean Towel (OP2) | 126 (72) | 606 (86.1) | 24 (14.2) | 125 (17.2) |
| Prepare Sterile Scissors / Blade (OP2) | 127 (72.6) | 583 (82.8) | 110 (65.1) | 551 (75.7) |
| Prepare Cord Ligature / Tie (OP2) | 175 (100) | 701 (99.6) | 168 (99.4) | 723 (99.3) |
| Prepare Mucus Extractor (OP2) | 172 (98.3) | 680 (96.6) | 165 (97.6) | 684 (94) |
| Prepare Bag & Mask (OP2) | 167 (95.4) | 668 (94.9) | 166 (98.2) | 717 (98.5) |
| Prepare Pads for Mother (OP2) | 170 (97.1) | 652 (92.6) | 91 (53.8) | 296 (40.7) |
| Checklist Used (OP2) | 87 (49.7) | 106 (15.1) | 0 (0) | 1 (0.1) |
| Observations at OP3 | 176 | 709 | 166 | 735 |
| Oxytocin Administered (OP3) | 150 (85.2) | 556 (78.4) | 48 (28.9) | 113 (15.4) |
| Other Uterotonic Administered (OP3) | 0 (0) | 0 (0) | 0 (0) | 0 (0) |
| Observations at OP4 | 171 | 699 | 178 | 754 |
| Baby Weight (OP4) | 137 (80.1) | 649 (92.8) | 138 (77.5) | 600 (79.6) |
| Baby Temperature (OP4) | 103 (60.2) | 249 (35.6) | 0 (0) | 1 (0.1) |
| Skin to Skin (OP4) | 126 (73.7) | 574 (82.1) | 5 (2.8) | 102 (13.5) |
| Skin to Skin 1 Hour (OP4) | 40 (23.4) | 136 (19.5) | 1 (0.6) | 1 (0.1) |
| Breastfeeding (OP4) | 128 (74.9) | 479 (68.5) | 10 (5.6) | 23 (3.1) |
| Checklist Used (OP4) | 139 (81.3) | 503 (72) | 0 (0) | 0 (0) |

OP=Observation Point
