# Supplementary material for: Nurses' and auxiliary nurse midwives' adherence to essential birth practices with peer coaching in Uttar Pradesh, India: a secondary analysis of the BetterBirth trial
Source: Implement Sci. 2020 Jan 3;15:1. doi: 10.1186/s13012-019-0962-7 (PMC6941293; doi:10.1186/s13012-019-0962-7)
Supplement: Supplementary file 3 — Additional file 3: Table S3. 6-month adherence to essential birth practices stratified by birth attendant cadre in 10 facilities in the BetterBirth trial. [file 13012_2019_962_MOESM3_ESM.docx]

**Additional file 3: Table S3. 6-month Adherence to Essential Birth Practices Stratified by Birth Attendant Cadre in 10 Facilities in the BetterBirth Trial**

|  | **Intervention** | | **Control** | |
| --- | --- | --- | --- | --- |
|  | **ANM** | **Staff Nurse** | **ANM** | **Staff Nurse** |
| Observations at OP1 | 57 | 318 | 12 | 263 |
| Mother's Temperature (OP1) | 16 (28.1) | 107 (33.6) | 0 (0) | 0 (0) |
| Mother's Blood Pressure (OP1) | 24 (42.1) | 173 (54.4) | 0 (0) | 0 (0) |
| Partograph Started (OP1) | 0 (0) | 3 (0.9) | 0 (0) | 0 (0) |
| Checklist Used (OP1) | 22 (38.6) | 166 (52.2) | 0 (0) | 0 (0) |
| Observations at OP2 | 62 | 312 | 11 | 254 |
| Oxytocin Administered (OP2) | 21 (33.9) | 122 (39.1) | 11 (100) | 239 (94.1) |
| Handwashing (OP2) | 0 (0) | 63 (20.2) | 0 (0) | 0 (0) |
| Prepare Clean Gloves (OP2) | 16 (25.8) | 278 (89.1) | 10 (90.9) | 205 (80.7) |
| Prepare Clean Towel (OP2) | 59 (95.2) | 300 (96.2) | 1 (9.1) | 100 (39.4) |
| Prepare Sterile Scissors / Blade (OP2) | 61 (98.4) | 306 (98.1) | 11 (100) | 248 (97.6) |
| Prepare Cord Ligature / Tie (OP2) | 62 (100) | 312 (100) | 11 (100) | 253 (99.6) |
| Prepare Mucus Extractor (OP2) | 62 (100) | 311 (99.7) | 11 (100) | 245 (96.5) |
| Prepare Bag & Mask (OP2) | 62 (100) | 312 (100) | 11 (100) | 198 (78) |
| Prepare Pads for Mother (OP2) | 61 (98.4) | 310 (99.4) | 11(100) | 200 (78.7) |
| Checklist Used (OP2) | 9 (14.5) | 40 (12.8) | 0 (0) | 1 (0.4) |
| Observations at OP3 | 59 | 316 | 13 | 251 |
| Oxytocin Administered (OP3) | 42 (71.2) | 230 (72.8) | 11 (84.6) | 56 (22.3) |
| Other Uterotonic Administered (OP3) | 0 (0) | 0 (0) | 0 (0) | 0 (0) |
| Observations at OP4 | 60 | 308 | 12 | 257 |
| Baby Weight (OP4) | 57 (95) | 298 (96.8) | 12 (100) | 221 (86) |
| Baby Temperature (OP4) | 12 (20) | 61 (19.8) | 0 (0) | 1 (0.4) |
| Skin to Skin (OP4) | 51 (85) | 223 (72.4) | 0 (0) | 2 (0.8) |
| Skin to Skin 1 Hour (OP4) | 0 (0) | 32 (10.4) | 0 (0) | 0 (0) |
| Breastfeeding (OP4) | 19 (31.7) | 171 (55.5) | 1 (8.3) | 3 (1.2) |
| Checklist Used (OP4) | 28 (46.7) | 247 (80.2) | 0 (0) | 0 (0) |

OP=Observation Point
