# Supplementary material for: Nurses' and auxiliary nurse midwives' adherence to essential birth practices with peer coaching in Uttar Pradesh, India: a secondary analysis of the BetterBirth trial
Source: Implement Sci. 2020 Jan 3;15:1. doi: 10.1186/s13012-019-0962-7 (PMC6941293; doi:10.1186/s13012-019-0962-7)
Supplement: Supplementary file 4 — Additional file 4: Table S4. 12-month adherence to essential birth practices stratified by birth attendant cadre in 30 facilities in the BetterBirth trial. [file 13012_2019_962_MOESM4_ESM.docx]

**Additional file 4: Table S4. 12-month Adherence to Essential Birth Practices Stratified by Birth Attendant Cadre in 30 Facilities in the BetterBirth Trial**

|  | **Intervention** | | **Control** | |
| --- | --- | --- | --- | --- |
|  | **ANM** | **Staff Nurse** | **ANM** | **Staff Nurse** |
| Observations at OP1 | 109 | 800 | 94 | 829 |
| Mother's Temperature (OP1) | 4 (3.7) | 126 (15.8) | 0 (0) | 0 (0) |
| Mother's Blood Pressure (OP1) | 10 (9.2) | 176 (22) | 0 (0) | 7 (0.8) |
| Partograph Started (OP1) | 0 (0) | 6 (0.8) | 0 (0) | 0 (0) |
| Checklist Used (OP1) | 25 (22.9) | 131 (16.4) | 0 (0) | 0 (0) |
| Observations at OP2 | 117 | 749 | 101 | 822 |
| Oxytocin Administered (OP2) | 69 (59) | 339 (45.3) | 64 (63.4) | 623 (75.8) |
| Misoprostol Administered (OP2) | 8 / 100 (8) | 47 / 613 (7.7) | 32 / 98 (32.7) | 118 / 665 (17.7) |
| Handwashing (OP2) | 2 (1.7) | 110 (14.7) | 0 (0) | 6 (0.7) |
| Prepare Clean Gloves (OP2) | 52 (44.4) | 603 (80.5) | 67 (66.3) | 668 (81.3) |
| Prepare Clean Towel (OP2) | 89 (76.1) | 497 (66.4) | 28 (27.7) | 268 (32.6) |
| Prepare Sterile Scissors / Blade (OP2) | 110 (94) | 745 (99.5) | 100 (99) | 798 (97.1) |
| Prepare Cord Ligature / Tie (OP2) | 115 (98.3) | 744 (99.3) | 101 (100) | 819 (99.6) |
| Prepare Mucus Extractor (OP2) | 110 (94) | 739 (98.7) | 95 (94.1) | 798 (97.1) |
| Prepare Bag & Mask (OP2) | 116 (99.1) | 749 (100) | 101 (100) | 817 (99.4) |
| Prepare Pads for Mother (OP2) | 85 (72.6) | 575 (76.8) | 17 (16.8) | 477 (58) |
| Checklist Used (OP2) | 7 (6) | 44 (5.9) | 0 (0) | 1 (0.1%) |
| Observations at OP3 | 110 | 746 | 99 | 820 |
| Oxytocin Administered (OP3) | 56 (50.9) | 431 (57.8) | 10 (10.1) | 114 (13.9) |
| Other Uterotonic Administered (OP3) | 6 (5.5) | 16 (2.1) | 7 (7.1) | 87 (10.6) |
| Appropriate Use of Bag & Mask (OP3) | 76 / 95 (80) | 565 / 608 (92.9) | 86 / 96 (89.6%) | 584 / 659 (88.6) |
| Observations at OP4 | 114 | 765 | 97 | 800 |
| Baby Weight (OP4) | 104 (91.2) | 727 (95) | 66 (68) | 678 (84.8) |
| Baby Temperature (OP4) | 5 (4.4) | 144 (18.8) | 0 (0) | 2 (0.3) |
| Skin to Skin (OP4) | 34 (29.8) | 544 (71.1) | 8 (8.2) | 55 (6.9) |
| Skin to Skin 1 Hour (OP4) | 4 (3.5) | 44 (5.8) | 0 (0) | 0 (0) |
| Breastfeeding (OP4) | 46 (40.4) | 278 (36.3) | 9 (9.3) | 34 (4.3) |
| Checklist Used (OP4) | 35 (30.7) | 271 (35.4) | 0 (0) | 0 (0) |

OP=Observation Point

Denominators reported where different from total.
