# Supplementary material for: Nurses' and auxiliary nurse midwives' adherence to essential birth practices with peer coaching in Uttar Pradesh, India: a secondary analysis of the BetterBirth trial
Source: Implement Sci. 2020 Jan 3;15:1. doi: 10.1186/s13012-019-0962-7 (PMC6941293; doi:10.1186/s13012-019-0962-7)
Supplement: Supplementary file 5 — Additional file 5: Table S5. Average percent adherence to essential birth practices by time point and birth attendant cadre. [file 13012_2019_962_MOESM5_ESM.docx]

**Additional file 5: Table S5. Average Percent Adherence to Essential Birth Practices by Time Point and Birth Attendant Cadre**

|  | **Intervention** | | | **Control** | |
| --- | --- | --- | --- | --- | --- |
|  | **ANM** | **Staff Nurse** | **p-Value*** | **ANM** | **Staff Nurse** |
| Baseline | 30.4 | 32.0 |  | 36.3 | 24.0 |
| 2 Months | 68.1 | 64.1 | 0.7617 | 33.8 | 33.2 |
| 6 Months | 56.2 | 62.0 |  | 40.7 | 35.9 |
| 12 Months | 49.2 | 56.1 | 0.6895 | 38.6 | 41.3 |

*This refers to comparison between ANM and Staff Nurse adherence to essential birth practices in the intervention arm at 2 months and 12 months after coaching started using logistic regression with Pearson over-dispersion correction.
